# Risk factors for posthepatectomy liver failure: a secondary analysis of prospective clinical trials

**DOI:** 10.1007/s00423-026-04107-1

**Published:** 2026-06-17

**Authors:** Alexander Betzler, Johanna Betzler, Christoph Reissfelder, Jürgen Weitz, Nuh N. Rahbari, Emrullah Birgin

**Affiliations:** 1 Department of Surgery, Medical Faculty Mannheim, University Hospital Mannheim, Heidelberg University, Mannheim, Germany; 2 Department of Visceral, Thoracic and Vascular Surgery, Faculty of Medicine Carl Gustav Carus, University Hospital, Technical University Dresden, Dresden, Germany; 3 Department of General and Visceral Surgery, University Hospital Ulm, Ulm University, 89081, Albert-Einstein-Allee 23, Ulm, Germany

**Keywords:** Posthepatectomy liver failure, ISGLS, Risk factors, Hepatectomy, Liver cirrhosis

## Abstract

**Background:**

Posthepatectomy liver failure (PHLF) remains the most lethal complication after liver resection. This study aimed to identify preoperative and intraoperative risk factors for PHLF following open hepatectomy using data derived from prospective clinical trials.

**Methods:**

A pooled secondary analysis of five prospective studies — four randomized controlled trials and one prospective cohort study — was performed, including 515 patients who underwent elective open liver resection between 2007 and 2017 at two high-volume hepatobiliary centers. PHLF was defined according to the International Study Group of Liver Surgery (ISGLS). Liver fibrosis, cirrhosis, and steatosis were assessed by histopathological evaluation of the surgical specimen. Risk factors were analyzed using univariable and multivariable logistic regression models.

**Results:**

PHLF occurred in 46 patients (9%), 9 Grade A (19.6%), 13 Grade B (28.3%), and 24 Grade C (52.2%) cases. The 90-day mortality rate in the PHLF group was 52.2%, compared to 2.1% in patients without PHLF. On multivariable logistic regression, liver cirrhosis (OR 4.23, 95% CI 1.82–9.84; *P* < 0.001), liver fibrosis (OR 2.16, 95% CI 1.01–4.62; *P* = 0.046), and major hepatectomy (OR 4.78, 95% CI 1.87–12.20; *P* = 0.001) were independently associated with PHLF. Patients with PHLF had significantly higher rates of severe complications (91.3% vs. 34.8%), bile leakage (45.7% vs. 18.6%), posthepatectomy hemorrhage (19.6% vs. 2.3%), and longer hospital stay (35 vs. 12 days).

**Conclusion:**

In this secondary analysis of prospective trials, cirrhosis, fibrosis, and major hepatectomy were found as independent risk factors for PHLF. Preventive strategies should emphasize careful patient selection, functional assessment, and parenchyma-sparing surgery.

**Clinical trial registration:**

Not applicable.

## Introduction

Posthepatectomy liver failure is the most fatal complication after liver surgery and characterized by impaired liver function with coagulopathy, hyperbilirubinemia, and clinical deterioration. The reported incidence of posthepatectomy liver failure (PHLF) varies, ranging from 1% to over 30%, depending on the extent of resection, patient comorbidities, and criteria used for diagnosis [1–4]. In the last decades, considerable progress in preoperative strategies aiming to mitigate the risk of PHLF has been witnessed in literature. Innovations such as portal vein embolization, associating liver partition and portal vein ligation for staged hepatectomy (ALPPS), and hepatic vein embolization have been developed to induce hypertrophy of the future liver remnant (FLR) prior to major resections [5, 6]. In addition, an increased adoption of parenchyma-sparing hepatectomy has decreased enormously rates of PHLF [7].

Despite these refinements in surgical techniques and perioperative care, the pathophysiology of PHLF remains multifactorial. Some risk factors were identified in previous cohort studies such as insufficient FLR volume, underlying chronic liver disease such as fibrosis or steatosis, preoperative chemotherapy-induced liver injury, intraoperative blood loss, and ischemia-reperfusion injury [8–12]. However, data from prospective trials are limited. While risk factors identified in retrospective series are broadly consistent, their confirmation within high-quality prospective datasets — with standardized operative techniques, prospective data recording, and use of International Study Group of Liver Surgery (ISGLS)-defined outcomes — remains scarce. Elucidating risk factors for PHLF within such a rigorous framework is critical for tailoring preoperative strategies, optimizing surgical planning, and improving patient outcomes. This study aims to analyze the incidence and predictors of PHLF using data derived exclusively from prospective clinical trials, thereby providing a level of evidence that has been lacking in this field.

## Materials and methods

A secondary data analysis was conducted in this study, using data from four randomized controlled trials (NCT00732979, NCT01049607, NCT01858987, NCT02612220) and one prospective cohort study (NCT01073345). These studies were carried out between April 2007 and September 2017 at two high-volume liver surgery centers - the Department of General, Visceral and Transplantation Surgery, University of Heidelberg, and the Department of Gastrointestinal, Thoracic and Vascular Surgery, University Hospital Carl Gustav Carus, Technical University Dresden [13–17]. The study was conducted in accordance with the Principles of Good Practice of Secondary Data Analysis (GPS) [18]. Ethical approval for this study was waived by the local institutional review board due to the use of de-identified data.

### Patient eligibility criteria and data extraction

The original publications included patient eligibility criteria for the individual trials. Patients who underwent liver resection and had available data on postoperative liver failure were included in the analysis. The present analysis extracted data from individual databases, including age, gender, body mass index (BMI), American Society of Anesthesiologists (ASA) score, diagnosis, presence of liver steatosis, fibrosis, cirrhosis, and perioperative blood values including bilirubin, aspartate aminotransferase (AST), alanine aminotransferase (ALT), alkaline phosphatase (AP), gamma glutamyltransferase (GGT) and international normalized ratio (INR). Operative details such as extent and type of resection, number of resected segments, and technique of hepatic parenchymal transection were also obtained.

## Definitions and outcomes

Posthepatectomy liver failure (PHLF) is defined according to the International Study Group for Liver Surgery (ISGLS) criteria, classifying PHLF into grades A, B, and C [4]. Grade A PHLF denotes postoperative hepatic dysfunction, presented with abnormal bilirubin or INR but without change of clinical management. Grade B PHLF refers to cases with noninvasive interventions, such as transfusions of fresh frozen plasma, treatment with albumin, diuretics, or noninvasive ventilation with transfer to intermediate or intensive care unit. Grade C PHLF requires invasive interventions, such as hemodialysis, mechanical ventilation, treatment with vasopressors or glucose infusion, extracorporeal liver support, or liver transplantation.

Liver fibrosis, cirrhosis, and steatosis were assessed by histopathological evaluation of the surgical specimen. Fibrosis was graded according to the Metavir scoring system (F0–F3), with F0 indicating no fibrosis and F3 indicating bridging fibrosis without cirrhosis. Liver cirrhosis was diagnosed histopathologically and classified according to the Child-Pugh scoring system (Child A and Child B). Hepatic steatosis was graded based on the proportion of hepatocytes containing macrovesicular fat vacuoles: Grade 1 (< 33%), Grade 2 (33–66%), and Grade 3 (> 66%).

The severity of postoperative complications was documented using the Clavien-Dindo classification, where clinically significant complications were defined as grade III or higher. Posthepatectomy bile leakage and posthepatectomy hemorrhage were recorded in line with the definitions by the ISGLS [19]. The analysis also considered variables and outcomes such as the need and duration of portal triad clamping, operating time, total blood loss, perioperative transfusion, postoperative hospital stays and mortality within 90 days after the surgery. Preoperative liver volumetry was conducted only in selected cases and resections were performed if the FLR was estimated ≥ 40%. Patients who had undergone preoperative portal vein embolization were not included in the study cohort.

## Operative techniques

Hepatic resections were performed in a standardized manner as described in the referenced study protocols [13–17]. In summary, all procedures were conducted via open laparotomy under conditions of low central venous pressure (CVP < 5 mmHg) to minimize intraoperative blood loss. Routine vascular inflow occlusion (e.g., Pringle maneuver) was not employed, except in cases of significant intraoperative hemorrhage, where it was used at the discretion of the operating surgeon. Parenchymal transection techniques included the clamp-crushing method, stapling devices, ultrasonic dissection (e.g., Cavitron Ultrasonic Surgical Aspirator CUSA), or the use of advanced energy sealing devices. Hemostasis was supported by the application of topical hemostatic agents and/or argon beam coagulation, utilized according to the individual surgeon’s preference. Inferior vena cava (IVC) clamping was performed selectively based on intraoperative judgment by the attending surgeon and anesthesiologist.

### Statistical analysis

Continuous data were reported as mean (SD) or median (IQR) and compared using the Student’s *t* test or the Wilcoxon rank-sum test, according to the distribution pattern. Categorical variables were described by absolute numbers and percentages, and group differences were assessed using the Chi-square test or Fisher’s exact test. Univariate logistic regression analysis was performed to identify risk factors associated with PHLF. Variables with a *P* value < 0.1 in univariate analysis were used for a multivariable analysis. Results were expressed as odds ratios (ORs) with corresponding 95% confidence intervals (CIs). A *P* value < 0.05 was considered statistically significant. Statistical analyses were performed using SPSS software (SPSS Inc).

## Results

### Patient characteristics

**Fig. 1:**
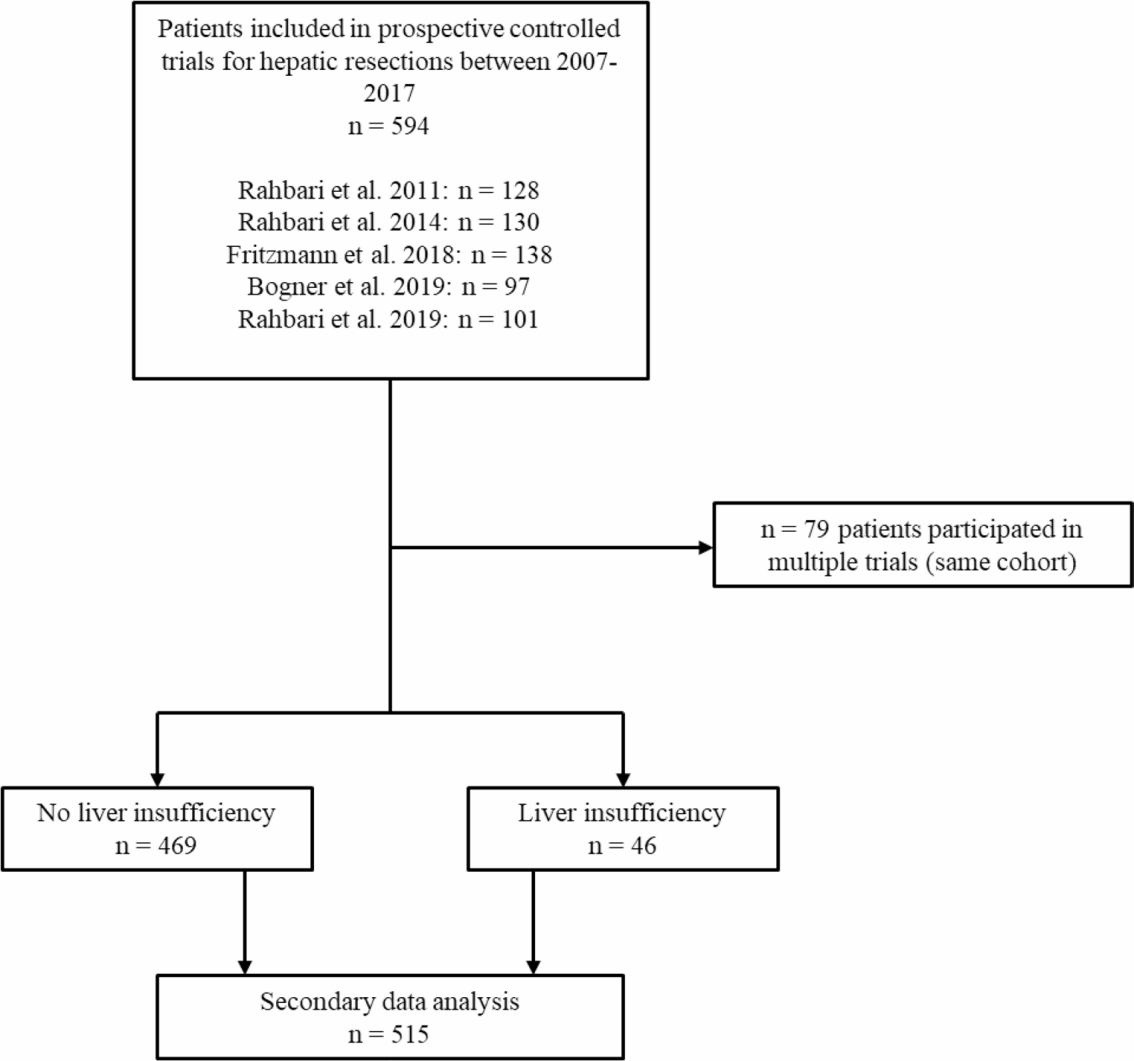
Study flow diagram

Of 594 patients included in the prospective controlled trials, a total of 515 patients met the inclusion criteria. Within this cohort, 46 (9.0%) patients experienced posthepatectomy liver failure, and 469 patients without experiencing PHLF served as control group. The study flow diagram is shown in Fig. 1.

There were 308 (59.8%) men and 207 (40.2%) women included (Table 1). Patients in the PHLF group were older (67 years [IQR: 62–73] vs. 62 years [IQR: 54–69], *P* = 0.001), had a higher proportion of hepatic fibrosis (69.6% vs. 48.0%; *P* = 0.018), and a higher prevalence of liver cirrhosis (30.4% vs. 10.5%; *P* < 0.001) compared to the control group. In the PHLF group most patients suffered from primary liver malignancies (65.2%), whereas in the control group the majority of patients had secondary liver malignancies (61.6%). Within the subgroup of patients with secondary malignancies, 215 of 289 patients (74.4%) in the non-PHLF group and 8 of 13 patients (61.5%) in the PHLF group received preoperative chemotherapy; this difference was not statistically significant (*P* = 0.365). There were no significant differences between groups with regard to ASA classification, preoperative BMI, or preoperative laboratory results.

**Table 1:** Baseline characteristics

	No liver failure (*n* = 469)	Liver failure (*n* = 46)	*P* value
Age (years) †	62 (54–69)	67 (62–73)	0.001
Gender			0.529
Male	278 (59.3%)	30 (65.2%)	
Female	191 (40.7%)	16 (34.8%)	
BMI (kg/m²) *	26 (5)	25 (4)	0.148
ASA			0.647
I	11 (2.3%)	0 (0.0%)	
II	196 (41.8%)	16 (34.8%)	
III	258 (55.0%)	30 (65.2%)	
IV	1 (0.2%)	0 (0.0%)	
Missing	3 (0.6%)	0 (0.0%)	
Steatosis			0.573
No	135 (28.8%)	16 (34.8%)	
Grade 1 + 2	311 (66.3%)	29 (63.0%)	
Grade 3	19 (4.1%)	1 (2.2%)	
Missing	4 (0.9%)	0 (0.0%)	
Fibrosis			**0.018**
No	242 (51.6%)	14 (30.4%)	
Grade 1 + 2	200 (42.6%)	25 (54.3%)	
Grade 3	25 (5.3%)	7 (15.2%)	
Missing	2 (0.4%)	0 (0.0%)	
Liver cirrhosis			**< 0.001**
No	420 (89.6%)	32 (69.6%)	
Child A	45 (9.6%)	12 (26.1%)	
Child B	4 (0.9%)	2 (4.3%)	
Diagnosis			**< 0.001**
Primary liver malignancy	135 (28.8%)	30 (65.2%)	
Secondary liver malignancy	289 (61.6%)	13 (28.3%)	
Benign liver disease	45 (9.6%)	3 (6.5%)	
Preoperative laboratory tests *			
Bilirubin (mg/dl)	0.83 (1.7)	0.93 (1.5)	0.717
AP (U/l)	152 (158)	159 (136)	0.793
GGT (U/l)	170 (270)	247 (356)	0.085
AST (U/l)	39 (36)	38 (29)	0.937
ALT (U/l)	43 (49)	38 (29)	0.537
Albumin (g/dl)	43 (3)	42 (5)	0.059
International normalized ratio	1.01 (0.1)	1.02 (0.1)	0.204
Preoperative chemotherapy ‡	215/289 (74.4%)	8/13 (61.5%)	0.365

Values in parentheses are percentages unless indicated otherwise; † Values are median (iqr); *Values are mean (s.d.); ‡ Preoperative chemotherapy reported for patients with secondary liver malignancy only (*n* = 302). ASA = American Society of Anesthesiologists, *BMI *body mass index, *AP* alkaline phosphatase, *GGT* gamma glutamyltransferase, *AST *aspartate aminotransferase, *ALT *alanine aminotransferase

## Operative details and posthepatectomy outcomes

Characteristics of surgery and posthepatectomy outcomes are summarized for both study groups in Table 2. In the PHLF group, there were 9 (19.6%) patients with Grade A PHLF, 13 (28.3%) patients with Grade B PHLF, and 24 (52.2%) patients with Grade C PHLF. Most patients with PHLF underwent right or extended right hepatectomies (67.4% vs. 30.9%). Major hepatectomy (87.0% vs. 51.8%; *P* < 0.001), vascular resections (21.7% vs. 6.6%; *P* = 0.002), longer operating time (275 min [IQR 194–362] vs. 179 min [IQR 136–240]; *P* < 0.001), higher blood loss (1400 ml [IQR 800–2175] vs. 800 ml [IQR 450–1400]; *P* < 0.001), with more frequent transfusions of packed red blood cells (41.3% vs. 14.9%; *P* < 0.001) and fresh frozen plasma (28.2% vs. 8.5%; *P* = 0.002) were more likely in the PHLF group compared to the control group.

**Table 2:** Operative details and postoperative outcomes

	No liver failure (*n* = 469)	Liver failure (*n* = 46)	*P* value
Extent of resection			< 0.001
Major hepatectomy	243 (51.8%)	40 (87.0%)	
Minor hepatectomy	226 (48.2%)	6 (13.0%)	
Type of resection			**< 0.001**
Right/extended right hemihepatectomy	145 (30.9%)	31 (67.4%)	
Left/extended left hemihepatectomy	87 (18.6%)	7 (15.2%)	
Central hepatectomy	8 (1.7%)	2 (4.3%)	
Anatomic resection > 2 segments	6 (1.3%)	0 (0.0%)	
Anatomic resection ≤ 2 segments	133 (28.4%)	4 (8.7%)	
Non-anatomical resection	110 (23.5%)	2 (4.3%)	
Extrahepatic resection	57 (12.2%)	11 (23.9%)	**0.039**
Vascular resection	31 (6.6%)	10 (21.7%)	**0.002**
Portal vein	11 (2.3%)	6 (13.0%)	
Hepatic artery	5 (1.1%)	0 (0.0%)	
Portal vein + hepatic artery	1 (0.2%)	2 (4.3%)	
Hepatic vein	5 (1.1%)	1 (2.2%)	
Inferior vena cava	9 (1.9%)	1 (2.2%)	
Pringle maneuver	99 (21.1%)	11 (23.9%)	0.706
IVC clamping	132 (28.1%)	15 (32.6%)	0.499
Operating time (min) †	179 (136–240)	275 (194–362)	**< 0.001**
Total blood loss (ml) †	800 (450–1400)	1400 (800–2175)	**< 0.001**
Intraoperative transfusion			
PRBCs †	70 (14.9%)	19 (41.3%)	**< 0.001**
FFP †	29 (8.5%)	11 (28.2%)	**0.002**
Posthepatectomy bile leak			**< 0.001**
Grade B	48 (10.2%)	13 (28.3%)	
Grade C	15 (3.2%)	5 (10.9%)	
Posthepatectomy hemorrhage			**< 0.001**
Grade B	1 (0.2%)	1 (2.2%)	
Grade C	4 (0.9%)	7 (15.2%)	
Clavien-Dindo classification			**< 0.001**
Grade I	40 (8.5%)	1 (2.2%)	
Grade II	70 (14.9%)	2 (4.3%)	
Grade IIIa	93 (19.8%)	4 (8.7%)	
Grade IIIb	41 (8.7%)	7 (15.2%)	
Grade IVa	19 (4.1%)	5 (10.9%)	
Grade IVb	0 (0.0%)	2 (4.3%)	
Grade V (death)	10 (2.1%)	24 (52.2%)	
Length of postoperative stay (days) †	12 (9–20)	35 (19–52)	**< 0.001**

Values in parentheses are percentages unless indicated otherwise; † Values are median (iqr); *Values are mean (s.d.); *IVC *inferior vena cava, *PRBC *packed red blood cell, *FFP *fresh frozen plasma, *MABP * mean arterial blood pressure, *SBP *systolic blood pressure

Bile leakage was significantly more frequent in the PHLF group compared to the control group (45.7% vs. 18.6%; *P* < 0.001), with a notably higher proportion of Grade B/C leakages (39.1% vs. 13.4%). Posthepatectomy hemorrhage was likewise observed more often in the PHLF group (19.6% vs. 2.3%; *P* < 0.001), with Grade C hemorrhage occurring particularly frequently (15.2% vs. 0.9%).

Patients with PHLF experienced a notably higher incidence of severe complications, defined as Clavien-Dindo grade III or above (91.3% vs. 34.8%; *P* < 0.001). As expected, the mortality rate in the PHLF group was also higher compared to the control group (52.2% vs. 2.1%; *P* < 0.001). The median duration of postoperative hospitalization was substantially longer among patients with PHLF (35 days [IQR 19–52] compared to 12 days [IQR 9–20]; *P* < 0.001).

## Risk factors for PHLF

In the next step, we performed logistic regression analyses including clinicopathological variables, particularly underlying pathological chronic liver disease, and intraoperative risk factors to identify potential risk factors associated with PHLF. Among the clinicopathological variables, liver cirrhosis emerged as the strongest independent predictor of PHLF, with an odds ratio (OR) of 4.23 (95% CI 1.82–9.84; *P* < 0.001), indicating a more than fourfold increased risk compared to patients without cirrhosis. Additionally, the presence of liver fibrosis was also independently associated with a higher likelihood of PHLF (OR 2.16, 95% CI 1.01–4.62; *P* = 0.046), whereas pathological diagnosis was not independently associated with PHLF. Patients undergoing major hepatectomies had a significantly higher likelihood of developing PHLF compared to those undergoing minor resections (OR 4.78, 95% CI 1.87–12.20; *P* = 0.001). Other operative variables such as the presence of extrahepatic disease, vascular resections, and combined extrahepatic resections were not found to increase the risk of PHLF (Table 3).

**Table 3:** Analysis of risk factors for PHLF

	UNIVARIATE		MULTIVARIABLE	
	OR (95%CI)	*P* value	OR (95%CI)	*P* value
Age (years)*	1.05 (1-1.1)	**0.001**	1.03 (1-1.1)	0.051
Diagnosis				
Primary Malignancy vs. Benign	3.33 (1-11.4)	0.056	0.9 (0.2–3.6)	0.885
Secondary Malignancy vs. Benign	0.68 (0.2–2.6)	0.551	0.45 (0.1–1.8)	0.25
Fibrosis				
Yes vs. No	2.46 (1.3–4.7)	**0.007**	2.16 (1.-4.6)	**0.046**
Liver cirrhosis				
Yes vs. No	4.19 (1.7–10.2)	**0.002**	4.23 (1.8–9.8)	**< 0.001**
Extent of hepatectomy				
Major vs. Minor hepatectomy	6.20 (2.6–14.9)	**< 0.001**	4.78 (1.9–12.2)	**0.001**
Extrahepatic resection				
Yes vs. No	2.23 (1.1–4.6)	**0.032**	2.14 (0.9-5)	0.079
Vascular resection				
Yes vs. No	3.92 (1.8–8.6)	**< 0.001**	1.96 (0.8–4.8)	0.147

*OR *odds ratio, *CI* confidence interval. * Per year increase

## Discussion

In this secondary analysis of five prospective trials including more than 500 patients undergoing open liver resection, we observed a PHLF rate of 9.0%. In addition, PHLF was identified as the most critical determinant of postoperative morbidity and mortality after hepatectomy, with a case-fatality rate exceeding 50% in affected patients. Patients who developed PHLF were more likely to present with hepatic fibrosis or cirrhosis, while major hepatectomy was identified as the strongest independent risk factor of PHLF. Our findings highlight the dual role of liver function and extent of resection as the major determinants of PHLF.

In the present study, liver cirrhosis was the strongest clinicopathological risk factor of PHLF, conferring more than a fourfold higher risk compared with patients without cirrhosis. Liver cirrhosis reduces functional reserve, impairs hepatic regeneration, and increases susceptibility to portal hypertension and intraoperative bleeding [20, 21]. Importantly, we also found that even the presence of fibrosis in non-cirrhotic patients doubled the risk of PHLF. This is consistent with experimental and clinical studies demonstrating that fibrotic remodeling compromises microvascular architecture and hepatocellular function, thereby limiting compensatory capacity after resection [22, 23]. These results emphasize that not only established cirrhosis, but also earlier stages of chronic liver disease should be recognized as significant risk factors. Therefore, accurate preoperative functional assessment and patient selection remain essential in clinical practice [24]. Cirrhotic patients often present with modifiable factors such as uncontrolled ascites, poor nutrition, or untreated viral hepatitis. Optimizing portal hypertension with non-selective beta-blockers, achieving viral suppression, improving nutritional status, and avoiding alcohol can enhance hepatic resilience [25, 26]. Evidence for pharmacologic strategies, such as the use of N-acetylcysteine, branched-chain amino acids, or antifibrotic therapies, is emerging but remains inconclusive [27–31]. Nonetheless, elective resections should be preceded by multidisciplinary optimization where possible.

The extent of resection remains a critical determinant, with major hepatectomy identified as the most significant surgical factor contributing to PHLF. The interplay between parenchyma quality and parenchyma loss emerges as a central concept: patients with cirrhosis or fibrosis who undergo major resections are at the highest risk. This finding is in line with numerous studies that have demonstrated exponential increases in PHLF incidence following major hepatectomy, particularly right or extended right hepatectomies [32, 33]. Despite demonstrated efficacy in predicting PHLF, preoperative volumetry is still underutilized in many institutions [34, 35]. Strategies to augment FLR such as portal vein embolization or associating liver partition and portal vein ligation for staged hepatectomy are established methods to reduce PHLF risk in patients requiring extended major hepatectomy [36, 37]. In our study, we excluded these patients with preoperative FLR modulation techniques, or liver remnants < 40%. The type of resection also influences outcomes, with the long-standing debate between anatomical resection and non-anatomical resection continuing to evolve. Anatomical resections have been associated with lower local recurrence in hepatocellular carcinoma by following portal territories [38]. However, some meta-analyses and propensity score studies demonstrated only modest or inconsistent survival advantages, while non-anatomic resections may be safer in compromised livers by sparing functional parenchyma [39, 40]. Our findings suggest that in patients with fibrosis or cirrhosis, the potential oncologic benefit of wider resections must be carefully weighed against the dramatically increased risk of PHLF. Individualized decision-making, incorporating tumor biology, liver quality, and volumetric assessments, is therefore essential.

The observed association between PHLF and other postoperative complications — particularly bile leakage and posthepatectomy hemorrhage — reflects a complex relationship. On the one hand, severe PHLF leads to coagulopathy and impaired immune defense, which may predispose both bile leakage and hemorrhage. On the other hand, significant blood loss and perioperative transfusions, both associated with major hepatectomy, may independently impair regenerative capacity, thereby contributing to PHLF. As the original prospective trials were not designed to analyze a temporal sequence of these events, causality cannot be established from the present data. The 90-day mortality rate in our cohort was 34 of 515 patients (6.6%), and 24 of the 34 deaths occurred in patients with Grade C PHLF, which is consistent with rates in current literature [41, 42]. Several cohort-specific factors contribute to this figure: a high proportion of major hepatectomies (52% overall), including patients with primary liver malignancies arising on a background of chronic liver disease, and the historical study period (2007–2017), during which enhanced recovery protocols were still evolving. The mortality rate among patients without PHLF was 2.1%, in line with benchmark data from high-volume hepatobiliary centers [43].

Minimally invasive approaches may also reduce the risk of PHLF [44]. Several studies have demonstrated that laparoscopic and robotic liver resections are associated with reduced blood loss, fewer transfusions, shorter hospital stays, and lower complication rates compared with open resections [45, 46]. Notably, some studies have suggested a lower incidence of PHLF with minimally invasive surgery, possibly due to improved visualization, reduced abdominal wall trauma, and preservation of collateral circulation [47, 48]. While our study included only open hepatectomies, future prospective trials directly comparing open and minimally invasive techniques in high-risk populations are warranted. Nevertheless, a major challenge in comparing outcomes across studies is the heterogeneity in definitions of PHLF. We applied standardized definitions as proposed by the International Study Group of Liver Surgery (ISGLS), which incorporate both biochemical and clinical parameters and allow grading of severity [49]. Importantly, ISGLS Grade A PHLF should not be considered clinically irrelevant, as recent studies link it to increased postoperative morbidity and interventions, despite a less consistent association with mortality [50, 51].

Yet, several series still rely on purely laboratory-based thresholds such as postoperative bilirubin levels or prothrombin time, leading to considerable variability in reported incidences, ranging from 1 to 30% [52–54]. Wider adoption of ISGLS criteria is essential for valid benchmarking and harmonization of clinical research as PHLF remains the major determinant of adverse outcomes after hepatectomy.

Our study has some limitations. As a secondary analysis of prospective trials performed at high-volume hepatobiliary centers, the reported rate of PHLF may be underestimated due to the high level of surgical expertise, multidisciplinary care, and established perioperative protocols. The studies did not collect distinct data on volumetry or hepatic venous-portal gradient measures, which are important predictors of PHLF. All resections were performed via open laparotomy, reflecting the trial designs of 2007–2017; this limits direct generalizability to contemporary minimally invasive practice, although the pathophysiological risk factors identified remain relevant regardless of the surgical approach. Furthermore, the patient populations enrolled in clinical trials may not fully reflect real-world practice, potentially introducing selection bias.

## Conclusion

PHLF continues to represent the Achilles’ heel of hepatic surgery, with devastating outcomes once severe failure occurs. Cirrhosis, fibrosis, and major hepatectomy are the dominant risk factors, emphasizing the importance of careful patient selection, functional testing, and parenchyma-sparing strategies. Preoperative optimization of cirrhosis, adoption of minimally invasive approaches where feasible, and centralization to high-volume centers may mitigate risk. Future research should focus on integrating functional liver tests into standardized preoperative algorithms, evaluating prehabilitation and antifibrotic therapies, and clarifying the role of minimally invasive surgery in preventing PHLF. Prevention remains the cornerstone of management, as therapeutic options for established PHLF remain limited and outcomes dismal.

## Data Availability

The data that support the findings of this study are available upon reasonable request.
